# Malvidin alleviates mitochondrial dysfunction and ROS accumulation through activating AMPK-α/UCP2 axis, thereby resisting inflammation and apoptosis in SAE mice

**DOI:** 10.3389/fphar.2022.1038802

**Published:** 2023-01-09

**Authors:** Panpan Zhao, Xiaomin Li, Qiankun Yang, Yingzhi Lu, Guanglu Wang, Haitao Yang, Jingquan Dong, Honggang Zhang

**Affiliations:** ^1^ Institute of Neuroscience, Department of Vascular Surgery, The First People’s Hospital of Lianyungang, Lianyungang, China; ^2^ Department of Oncology, The Second People’s Hospital of Lianyungang City, Lianyungang, China; ^3^ Jiangsu Key Laboratory of Marine Bioresources and Environment, Co-Innovation Center of Jiangsu Marine Bio-industry Technology, Jiangsu Key Laboratory of Marine Pharmaceutical Compound Screening, College of Pharmacy, Jiangsu Ocean University, Lianyungang, China

**Keywords:** malvidin, mitochondrial dysfunction, ROS, AMPK-α, UCP2, sepsis-associated encephalopathy

## Abstract

This study aimed to explore the protective roles of malvidin in life-threatened sepsis-associated encephalopathy (SAE) and illustrate the underlying mechanism. SAE mice models were developed and treated with malvidin for subsequently protective effects evaluation. Malvidin restored neurobehavioral retardation, declined serum S100β and NSE levels, sustained cerebrum morphological structure, improved blood-brain barrier integrity with elevated tight junction proteins, and decreased evans blue leakage, and finally protect SAE mice from brain injury. Mechanistically, malvidin prevented cerebrum from mitochondrial dysfunction with enhanced JC-1 aggregates and ATP levels, and ROS accumulation with decreased lipid peroxidation and increased antioxidant enzymes. UCP2 protein levels were found to be decreased after LPS stimulation in the cerebrum and BV-2 cells, and malvidin recovered its levels in a ROS dependent manner. *In vivo* inhibition of UCP2 with genipin or *in vitro* interference with siRNA UCP2 both disrupted the mitochondrial membrane potential, decreased ATP levels and intensified DCF signals, being a key target for malvidin. Moreover, dorsomorphin block assays verified that malvidin upregulated UCP2 expression through phosphorylating AMPK in SAE models. Also, malvidin alleviated SAE progression through inhibition of ROS-dependent NLRP3 inflammasome activation mediated serum pro-inflammatory cytokines secretion and mitochondrial pathway mediated apoptosis with weakened apoptosis body formation and tunel positive signals, and decreased Bax, cytochrome C, caspase-3 and increased Bcl-2 protein levels. Overall, this study illustrated that malvidin targeted AMPK-α/UCP2 axis to restore LPS-induced mitochondrial dysfunction and alleviate ROS accumulation, which further inhibits NLRP3 inflammasome activation and mitochondrial apoptosis in a ROS dependent way, and ultimately protected SAE mice, providing a reference for the targeted development of SAE prophylactic approach.

## Introduction

Sepsis, a serious clinical syndrome in the field of critical care, is a life-threatening multi-organ dysfunction caused by an uncontrolled acute systemic inflammatory response of the host to bacterial toxins and has become a major public health problem worldwide (Singer et al., 2016). The central nervous system (CNS) is considered to be one of the organs affected in the early stages of sepsis, which manifests clinically as sepsis-associated encephalopathy (SAE), with approximately 70% of sepsis patients exhibiting SAE symptoms (Gofton and Young, 2012). SAE manifests as diffuse brain dysfunction and cognitive dysfunction, and is positively associated with mortality in sepsis patients (Widmann and Heneka, 2014; Helbing et al., 2018). It is reported that SAE patients have a mortality rate of 70%, while about 10%–20% of SAE survivors may develop long-term neurocognitive deficits, psychiatric disorders, and neurodegenerative diseases (Annane and Sharshar, 2015; Heming et al., 2017). To date, SAE remains a pressing clinical challenge, and its pathogenesis is not yet fully understood, resulting in no effective prevention and treatment methods. Therefore, it is necessary to actively explore the pathogenesis of SAE and find effective drugs to reduce the incidence and severity of septic brain injury.

Mitochondria are the material center of cell synthesis and metabolism, known as the “power plant” of the cell. It is mainly involved in regulating cellular energy metabolism ATP production and reactive oxygen species (ROS) generation (Annesley and Fisher, 2019), as well as maintaining calcium homeostasis and endogenous apoptosis and autophagy (Cogliati et al., 2016). Several studies have suggested that mitochondrial dysfunction mediated energy metabolism disruption, reduced antioxidant production, and free radical accumulation are the main causes of neurological deficits and eventual development of SAE in patients with sepsis, as well as an important pathophysiological basis for SAE (Sharshar et al., 2003; Messaris et al., 2004). The brain consumes a large amount of energy and oxygen, and operates mainly on the energy derived from oxygen intake from the circulation and the conversion of glucose in the mitochondria. Therefore, once the mitochondria become dysfunctional, the impact on the brain tissue is particularly prominent, which eventually leads to a series of clinical manifestations of neurological abnormalities (d'Avila et al., 2008). Mitochondrial dysfunction triggered ROS accumulation is thought to be a activation signal for NLRP3 inflammasome. The innate immune system is the host’s first defense line against infection, and intracellular NLRP3 is capable of sensing multiple stimulus signals involved in the immune regulation of diseases. ROS stimulation mediates NLRP3 oligomerization with ASC, recruitment of inflammatory caspases, assembly of inflammasomes to produce activated caspases, cleavage of IL-1β precursors into mature IL-1β, and mediation of inflammatory responses (Kelley et al., 2019). Apoptosis is a kind of programmed cell death and is involved in the development of sepsis and SAE (Cao et al., 2019; Zhou et al., 2020). ROS accumulation promotes apoptosis and causes mitochondrial membrane potential changes, leading to the translocation of Bax and Bad across the mitochondrial membrane, and release of cytochrome c (Circu and Aw, 2010). The release of cytochrome c into the cytoplasm and caspase-9-mediated caspase-3 activation mediates the mitochondria pathway apoptosis (Estaquier et al., 2012). Thus, maintaining mitochondrial function and ROS homeostasis may play important roles in regulating SAE. Finding drugs that target the regulation of mitochondrial function and ROS production may become effective strategies for the treatment of SAE.

Uncoupling proteins (UCPs) are located in the inner mitochondrial membrane and influence mitochondrial function and body metabolism through uncoupling of oxidative phosphorylation. The mammalian genome encodes five UCP homologues, UCP1-5 (Krauss et al., 2005). Among them, UCP2 is the most widely distributed, commonly found in the CNS, kidney, heart, liver, pancreas, spleen, brain, skeletal muscle, thymus and macrophages (Ricquier et al., 1999), regulating mitochondrial membrane potential and the production of ROS (Toda and Diano, 2014). Previous studies showed that UCP2 regulated the production of ROS through the regulation of mitochondrial synthesis and fatty acid oxidation to inhibit the inflammatory response and the development of apoptosis (Deng et al., 2012; Zhong et al., 2019). AMPK is an evolutionarily conserved stress-sensing protein kinase, that is, activated to maintain cellular metabolic homeostasis by sensing a decrease in ATP (Rabinovitch et al., 2017). The activation of UCP2 may be associated with a related pathway that relies on its upstream AMPK-mediated antioxidant response (Rabinovitch et al., 2017; Huang et al., 2019; Yu et al., 2019). In sepsis-related injury models, UCP2 exerts protective functions in heart (Chen et al., 2018), lung, spleen injury (Jabůrek et al., 2018), but adverse functions in liver injury (Le Minh et al., 2009). However, in a sepsis-associated astrocyte model, UCP2 was found to protect mitochondria, which may play a protective role in SAE (Peng et al., 2019). Therefore, it is urgently needed to search for drugs that target the regulation of UCP2 protein expression and thus maintain homeostasis of mitochondrial function and ROS production in the treatment of SAE.

Flavonoids are the most common active polyphenols, widely distributed in plants, with a variety of pharmacological effects, including anti-inflammatory (Hussain et al., 2016), anti-cancer (Kopustinskiene et al., 2020), anti-proliferative (Fernández et al., 2021), antioxidant, and inhibition of free radical-mediated processes (Parhiz et al., 2015). Many studies have reported that flavonoids alleviate sepsis-induced brain injury (Zhu et al., 2019), memory impairment (Gong et al., 2019), cognitive dysfunction (Karimian, 2020), and sepsis-induced lung damage (Toklu et al., 2008), kidney damage (Ren et al., 2020), and heart damage (Wu B. et al., 2020) through antioxidant and anti-inflammatory functions. Anthocyanins are a group of flavonoids with similar anti-inflammatory and antioxidant properties to other members of flavonoids family (Aqil et al., 2012). Malvidin is the most common anthocyanin with antioxidant, anti-inflammatory, antibacterial, analgesic effects and anticholinesterase activity (Prudente et al., 2013). Lipopolysaccharides (LPS) can indirectly cross the blood-brain barrier into the brain, thus simulating SAE model. It has been shown that malvidin can alleviate oxidative stress and inflammation in LPS stimulated human peripheral blood mononuclear cells (Bastin et al., 2020), human THP-1 cells (Bastin et al., 2021) and other cell types. However, whether malvidin can protect against septic brain injury and the underlying mechanisms are not clear.

Systemic inflammation induced by lipopolysaccharide will activate inflammatory responses in the brain (Gofton and Young, 2012; Singer et al., 2016). Microglia, a primary innate immune cell in the CNS, are the first defense line against infection and brain tissue injury. Upon sensing signals from exogenous stimuli, the resting microglia cells are activated to interact with pathogens or exogenous ligands to induce neuroinflammation and a variety of signal transmission. Microglia play an important role in communication between systemic inflammation and the CNS (Cunningham, 2013). And BV2 cell line is an ideal *in vitro* model for SAE study (Joshi et al., 2021). The present study investigated the protective effects and potential mechanisms of malvidin in LPS-induced SAE mice and BV-2 cells neuroinflammation models, and proposed the following hypothesis: malvidin targets AMPK-α/UCP2 axis to restore LPS-induced mitochondrial dysfunction and alleviate ROS accumulation, which further inhibits ROS-mediated NLRP3 inflammasome activation and mitochondrial apoptosis, and ultimately alleviate cerebrum injury in LPS induced SAE model. The aim of this study was to elucidate the mechanism of SAE occurrence and the protective role of malvidin in SAE, and to provide a reference for the targeted development of SAE prophylactic approach.

## Material and methods

### Mice and experimental grouping

Male BALB/c mice (6-week-old, 20–25 g weight) were provided by Pizhou Oriental (Pizhou, China), and housed in plastic cages (6 mice in 290 × 178 × 160 cm size) to acclimatize the environment conditions (25°C ± 2°C room temperature, 55 ± 15% humidity, 12 h light/dark cycles) for 7 days with adequate amount of drinking water and food. The mice procedures were strict adherence to the “Jiangsu Ocean University Animal Welfare and Research Ethics Committee guidelines”, the “Principles of Laboratory Animal Care” (NIH publication No. 85–23, revised 1985) and NIPRD’s standard operation procedures (NIPRD/05.03.05–1).

Sixty mice were averagely divided into five groups with 12 mice in each group, including negative control group, LPS injection group (dissolved in 0.9% NaCl solution, 10 mg/kg body weight, BW, *Escherichia coli* O111:B4 strain, Sigma-Aldrich, MO, United States), LPS injection combined with malvidin (dissolved in DMSO, 5, 10, and 20 mg/kg BW, CAS# 643-84-5, Yuanye Biotechnology Ltd., Shanghai) treatment groups. Mice in the malvidin treatment groups were given drugs *via* intraperitoneal injection (i.p.) once a day for 7 days, whereas the other two groups were treated with equal volume of 0.9% NaCl solution. All mice were injected with LPS at the 4th day except for the control group and sacrificed after 6 h since the last malvidin injection on the 7th day. Blood and cerebrum samples were isolated for further assays. No mice were dead during the experimental period.

For UCP2 inhibitor block *in vivo*, four genipin (Selleck, Shanghai) experimental groups with six mice in each group were established. Mice were gavaged with genipin (100 mg/kg BW, Selleck, Shanghai) once a day for 3 days before LPS injection (Ding et al., 2019), and only genipin treatment mice were used as control.

### Neurological reflex scoring

Before mice sacrifice, a series of neurological reflex evaluation tests, including auricular reflex, corneal reflex, turn-right reflex, tail-flick reflex and escape reflex, were conducted to evaluate whether the SAE model was successfully established. For the auricular reflex test assessment, rub the ear canal of the mouse ear with a wooden stick to elicit head shaking. For the corneal reflex test assessment, gently touch the cornea with a cotton swab to elicit head shaking or blinking. For the turn-right reflex test assessment, turn the mouse to supine position and wait for it to return to prone position on its own. For the tail-flick reflex test assessment, briefly pinch the tail to observe if it elicits a ducking response. For the escape reflex test assessment, give a brief stimulus to the carapace to observe if it elicits an escape stimulus. For the escape reflex test assessment, give a brief stimulus to the body to see if it causes an escape stimulus. The above reflexes were recorded as two points if they occurred within 1 s, one point if they occurred within 1–10 s, and 0 point if they did not. The total score of each group of mice was calculated, and a score of no more than six was considered successful for SAE models. All the neurological reflex assays were performed under blinded tests.

### Cell culturing, transfection and stimulation

Murine microglial cell line BV-2 was obtained from the Institute of Basic Medical Sciences, Chinese Academy of Medical Sciences (Beijing, China) and cultured in 10% DMEM medium (Servicebio, Wuhan, China) supplemented with 10% FBS (Biological Industries, Israel) and 1% penicillin and streptomycin solution at 37°C/5% CO_2_. BV-2 cells were seeded into 96-well cell culturing plates and incubated with malvidin (10, 50, 100, 200, and 500 μM), LPS (10, 50, 100, 500, and 1000 ng/ml), or malvidin plus 500 ng/ml LPS for 24 h. The cell viability was determined under OD_450nm_ using Cell Counting Kit-8 (CCK-8, Beyotime) referring to the instructions.

For inhibition of UCP2, BV-2 cells were transiently transfected with small interfering RNA targeting to UCP2 (si-UCP2, 5′-GCU​AAA​GUC​CGG​UUA​CAG​ATT-3′, General Biol, Anhui, China) (Dando et al., 2017) using Lipofectamine 3000 transfection reagent (Invitrogen, United States) referring to the instructions. After 48 h, cells were incubated with malvidin followed by LPS stimulation. The single siRNA negative control (si-NC, 5′-CAG​UCG​CGU​UUG​CGA​CUG​G-3′) treatment or single si-UCP2 treatment groups were used as control groups.

For inhibition of AMP-activated protein kinase α (AMPKα), BV-2 cells were pretreated with dorsomorphin (10 μM, MCE, Shanghai) for 2 h (Chen et al., 2019) and then incubated with malvidin followed by LPS stimulation. For activation of NLRP3 inflammasome *in vitro*, BV-2 cells were stimulated with LPS for 4 h and 5 mM ATP for 30 min after malvidin treatment for 2 h. For inhibition of ROS *in vitro*, BV-2 cells were pretreated with 10 μM N-acetylcysteine (NAC, Selleck) for 2 h followed by malvidin treatment for 2 h and 500 ng/ml (NLRP3 inflammasome assays) or 1 μg/ml (apoptosis assays) LPS stimulation for 24 h.

### Enzyme linked immunosorbent assay (ELISA)

Mice serum was isolated from fresh blood samples after placing at 4°C overnight and centrifuging at 1,000 × g for 20 min. The concentrations of mouse S100 calcium binding protein β (S100β) and neuron specific enolase (NSE) were determined by ELISA kits (Elabscience, Wuhan, China) according to the guided instructions. The optical density values were measured at 450 nm on a multifunctional full wavelength microplate reader (Bioteck, Beijing, China), and the concentrations of S100β and NSE were determined using each established standard curves.

Serum or cell culturing supernatants were collected for inflammatory cell cytokines of IL-1β, IL-6, TNF-α secretion levels determination using corresponding ELISA detection kits (Invitrogen, United States) referring to the instructions. The cytokines concentrations were calculated using each standard curves.

### Histopathological observation

The cerebral cortex area was cut into small patch and placed in 10 times the volume of tissue fixative fluid (Biosharp, Beijing, China) for 12 h. The tissues were then dehydrated in alcohol, cleared in xylene, embedded in the mixture of xylene and paraffin, and cut into 4-μm thick after solidification and freezing treatment. Next, the slices were dewaxed in xylene, hydrated in alcohol and distilled water, and stained with hematoxylin & eosin (HE). The histopathological morphology was observed under blinded tests and typical changes were evaluated by pathologists.

### Immunofluorescence

Fresh cerebrum tissues were cut into small patches and made into 20 μm thick frozen sections. Then, the slices were fixed in tissue cell fixation fluid, permeabilized in 0.2% Triton X-100, and washed using PBS. The slices were incubated with primary antibody of IBA1 antibody (1:100, Proteintech, Wuhan, China) at 4°C overnight and detection antibody of fluorescein-conjugated goat anti-rabbit IgG (1:400, Proteintech) at 37°C for 1 h. The cell nucleu was stained DAPI (2 μg/ml) at room temperature for 5 min. The IBA1 labeled activated glial cells were observed on a laser scanning confocal microscope (LSCM, Nikon A1, Tokyo).

### Real-time fluorescence quantitative PCR (RT-qPCR)

RNA was extracted from fresh cerebrum cortex tissue samples using Trizol reagent (Vazyme, Nanjing, China). After removal of genomic DNA contamination, cDNA was prepared using MonScript RTIII Super Mix (Monad, Wuhan, China). RT-qPCR assays was carried out with 10 times diluted cDNA and amplified each targets with specific primer sets using SYBR Green qPCR mix (Monad). The targets mRNA relative levels were determined using 2^−ΔΔCT^ method. The primer sets information were shown in Supplementary Table S1.

### Evans blue leakage

Prior to sacrifice, mice were injected i. p. with 0.5% evans blue (dissolved in 0.9% NaCl solution, 80 mg/kg BW, Sigma, United States). After 2 h, mice were transcardially perfused with ice-phosphate buffer saline (PBS) and cerebrum tissue samples were quickly collected and weighted. The cerebrum tissue were fully homogenized in 1 ml PBS and centrifuged at 15,000 × g for 30 min. The supernatants were mixed with equal volume of trichloroacetic acid and incubated at room temperature for 24 h. After centrifugation, the supernatants were used for optical density values measurement at 620 nm. The evans blue leakage into the cerebrum was quantified by the established standard curve (Manaenko et al., 2011). Meanwhile, fresh cerebrum tissues were cut into small patches and made into 20-μm thick frozen slices. The evans blue fluorescence signal were observed on a confocal microscope (Olympus, Japan) under 405 nm.

### Mitochondrial purification and membrane potential (MMP) determination

Mice cerebrum mitochondria was isolated using Tissue Mitochondria Isolation Kit (Beyotime, Beijing, China) according to the manufacture’s instructions. In detail, fresh cerebrum tissue was mixed with 10 times the volume of pre-cooled mitochondrial isolation reagent, and homogenized thoroughly on ice. Then, the supernatants were collected by centrifugation at 1,000 × g for 5 min to remove intact cell debris and nuclei. The purified mitochondrial precipitates were isolated from supernatants through further centrifugation under conditions of 11,000 × g for 10 min, and dissolved in mitochondrial storage solution.

The MMP of purified mitochondria was determined using Mitochondrial membrane potential assay kit (Beyotime) with JC-1 fluorescence probe. Briefly, mitochondria were mixed with 9 times the volume of JC-1 staining solution and the fluorescence intensity of JC-1 aggregates was detected on a multifunctional full wavelength microplate reader at the excitation and emission wavelength of 485 nm and 590 nm, respectively.

To evaluate the MMP changes in BV-2 cells, cells were stained with JC-1 staining solution at 37°C/5% CO_2_ for 20 min and observed on a LSCM. The JC-1 monomers signals were detected at the excitation and emission wavelength of 488 nm and 525 nm, whereas the JC-1 aggregates were measured at the excitation and emission wavelength of 540 nm and 590 nm. Images were captured by NIS-elements Viewer Imaging software. The MMP changes were calculated through measuring the ratio of JC-1 aggregates in the experimental groups and control group.

### ATP contents

ATP contents were measured in purified cerebrum mitochondrial and cerebrum tissue homogenates with ATP Content Assay Kit (Beyotime) according to the instruction manual operation steps. Specially, the cerebrum tissue homogenates in ATP measurement assay were prepared as the following. Fresh cerebrum tissues were mixed with 10 times the volume of the extract solution, homogenized on ice, and the supernatant was collected after centrifugation at 12,000 × g for 10 min. The absorbance value of each reaction mixture were determined on a UV spectrophotometer at 340 nm wavelength.

### ROS levels

The ROS levels in the purified cerebrum tissue mitochondrial or BV-2 cells were detected using Reactive Oxygen Species Assay Kit (Biosharp) with fluorescent probe H_2_DCFDA. The cerebrum tissue mitochondrial or BV-2 cells were incubated with H_2_DCFDA to a final concentration of 10 μM under conditions of 37°C for 30 min in the dark. The fluorescence intensity in the cerebrum tissue homogenate was detected on a multifunctional full wavelength microplate reader, whereas the fluorescence signals in the BV-2 cells were captured using the LSCM at the excitation and emission wavelength of 488 nm and 525 nm, respectively.

### Biochemical indexes testing

Peroxidation indicator of malondialdehyde (MDA) and antioxidant indicators of superoxide dismutase (SOD), glutathione peroxidase (GSH-Px), catalase (CAT) in cerebrum tissue homogenate were tested using corresponding biochemical kits (Jiancheng, Nanjing, China) referring to the instructions. Specially, the protein concentrations in cerebrum tissue homogenate were quantified using BCA method with Pierce BCA Protein Quantification Kit (Thermo Scientific, United States) according to the instructions. Lactate dehydrogenase (LDH) levels in the serum were detected on an automated analyzer (Ortho, United States) referring to the instructions using LDH cytotoxicity assay kit (Beyotime, Beijing, China) referring to the instructions.

### Transmission electron microscopy (TEM) observation

Fresh cerebrum tissue samples were isolated and cut into small patches. The patches were fixed in 2.5% glutaraldehyde fixative (pH = 7.3) at 4°C overnight, dehydrated in acetone, resin soaked overnight at room temperature, polymerized at high temperature, sectioned with a slicing machine (Leica, Germany) to 70 nm thickness. Then, the slices were stained with uranyl acetate for 15 min and lead citrate for 10 min, dried and placed on a carbon-supported film copper grid, and observed by TEM (Hitachi H-7650, Japan) for observation.

### Protein electrophoresis and immunoblotting

Cerebrum tissue or BV-2 cell protein samples were extracted using RIPA buffer (Solarbio, Beijing, China) supplemented with 1 mM phenyl-methylsulfonyl fluoride (PMSF) according to the instructions, and quantified with Pierce BCA Protein Quantification Kit using BSA standards. Equal amount protein (20 μg) were separated through electrophoresis on 15% SDS-PAGE gels and transferred onto PVDF membranes (Mollopire, MA, United States). Specially, the membranes were incubated with primary antibodies of UCP2 (Wanleibio), Phospho-AMPKα (Wanleibio), NLRP3 (Adipogen, Liestal, Switzerland), Caspase-1 (Adipogen), IL-1β (R&D, United States), Bax (Proteintech), Bcl-2 (Proteintech), cytochrome C (Cyto-C, Proteintech), caspase-3 (Wanlei) or GAPDH (Proteintech), and secondary antibodies of HRP-conjugated goat anti-mouse, goat anti-rabbit or rabbit anti-goat IgG (Proteintech). The membranes were developed with ECL chemiluminescent solution (Vazyme, NanJing, China) and imaged *via* a multifunctional ultra-sensitive imaging system (SHST, Shanghai, China).

### Tunel staining

Apoptosis analysis in the cerebrum tissue paraffin slices was characterized using a TUNEL BrightRed Apoptosis Detection Kit (Vazyme, Nanjing, China) referring to the instructions. The cell nuclei was stained with Hoechst 33258 (UE, Suzhou, China).

### Statistical analysis

Graphs were presented as mean ± standard deviation (SD) using GraphPad Prism software version 8.0.1 (224). Statistical analysis was conducted using one-way analysis of variance (ANOVA) followed by Tukey’s *post hoc* testing using SPSS software version 26. Differences were considered statistically significant when the *p* values was less than 0.05.

## Results

### Protective roles of malvidin on LPS-induced cerebrum injury

**FIGURE 1:**
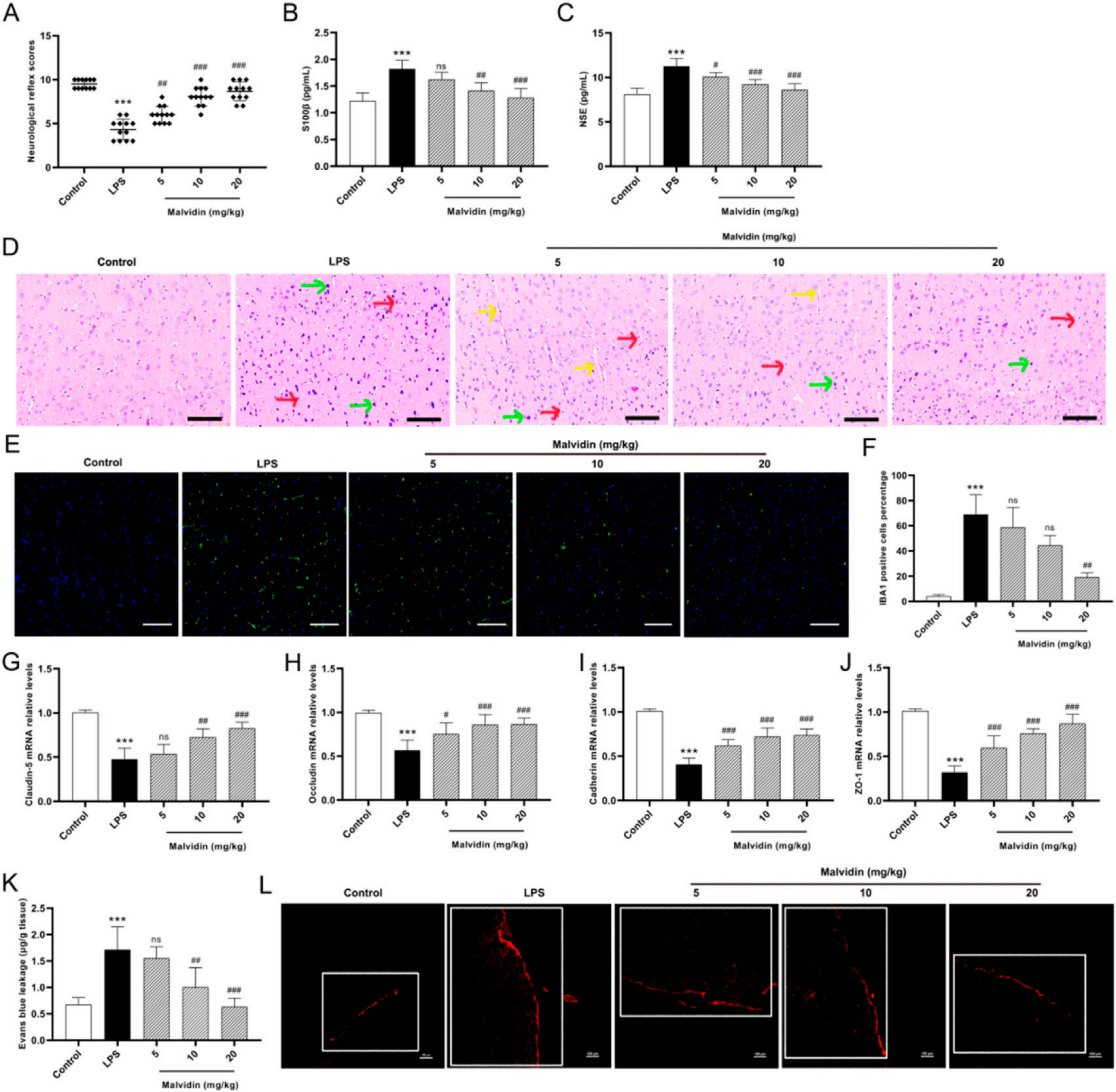
Effects of malvidin on LPS-induced mice cerebrum injury. **(A)** Scores of neurological reflex indicators of auricular reflex, corneal reflex, turn-right reflex, tail-flick reflex and escape reflex (*n* = 12). **(B–C)** Serum S100β and NSE levels analysis using ELISA assays (*n* = 6). **(D)** Histopathological changes observations of cerebrum cortex using H&E staining (*n* = 6). The red arrows represent neuronal necrosis, green arrows represent glial cells, and yellow arrows represent capillary dilation. **(E)** Immunofluorescence observation of glial cells activation in the cerebrum. The green fluorescence signals are IBA1 positive cells, whereas the blue signals are cell nucleus. **(F)** Quantitative analysis of IBA1 positive cells percentage in cerebrum tissues (*n* = 3). **(G–J)** The mRNA relative expression levels analysis of tight junction proteins of ZO-1, claudin-5, occludin, and cadherin using RT-qPCR analysis (*n* = 6). **(K)** Quantitative analysis of evans blue leakage in cerebrum tissues under optical density values of 620 nm (*n* = 6). **(L)** Confocal microscope observation of evans blue leakage in the cerebrum cortex (white box). The red fluorescence signals are evans blue. Scale bar = 100 μm * represents comparison between LPS group and control group, whereas # indicates analysis among LPS group and malvidin pretreatment groups. # is *p* < 0.05, ## is *p* < 0.01, ***/### is *p* < 0.001, and ns is *p* > 0.05.

To assess the protective effect of malvidin on LPS-induced cerebrum injury, neurological reflex scoring tests were first conducted. Prior to sacrifice, mice were utilized to test five neurological behavior assays, including auricular reflex, corneal reflex, turn-right reflex, tail-flick reflex and escape reflex. The results showed that the neurological reflex scores of mice in the LPS-injected group (mean value of 4.33) was significantly lower than that of the control group (mean value of 9.5), indicating the successful establishment of a mouse pathological model of SAE. Under treatment with malvidin, the low dose could increase the neurological reflex scores (mean value of 6) but the difference was not significant (*p* > 0.05), and the medium and high doses could significantly restore the neurobehavior of mice (*p* < 0.001, mean values of 8.08 and 8.67, respectively). These data indicates that malvidin can restore neurobehavioral retardation caused by septic cerebrum injury (Figure 1A). S100β and NSE are widely recognized as biomarkers of central nervous system injury (Kawata et al., 2016). Serum S100β and NSE levels may indirectly reflect the extent of damage in SAE (Wu L. et al., 2020). The results of serum ELISA showed that LPS injection significantly enhanced the release levels of S100β and NSE, while malvidin treatment dose-dependently declined their release levels, thus reducing the degree of nerve damage (Figures 1B, C). Histopathological observations showed that LPS injection caused most of the neurons in the cerebral cortex to be solidified and deeply stained, with an increase in glial cells compared to the control group. Only individual neuronal fixation and deep staining were observed after low and medium doses of malvidin treatment, but accompanied by pathological changes of capillary dilation. In contrast, the morphological structure of the cerebral cortex was normalized after high-dose malvidin pretreatment, and only individual neuronal cells were solidified and deeply stained, but glial cells still showed an increase compared with the control group (Figure 1D). Immunofluorescence results further indicated that glial cells were activated after LPS injection, and the activated glial cells significantly decreased when treatment with high dose of malvidin (Figures 1E, F). These results suggest that malvidin can protect cerebrum neurons against LPS-induced neuronal necrosis. Blood-brain barrier (BBB) disruption is an important indicator of SAE. Tight junction (TJ) proteins maintain BBB integrity (Peng et al., 2021). Therefore, we examined several key TJ expression profiles. The results showed that LPS injection caused a significant decrease in the transcript levels of ZO-1, claudin-5, occludin, and cadherin compared to the control group, while malvidin dose-dependently increased the transcript levels of these genes, thereby restoring BBB integrity (Figures 1G–J). To further confirm our results, evans blue leakage experiments were used to observe the evans blue content into the BBB. We first quantified evans blue in the cerebrum and showed that LPS injection caused a significant increase in the amount of evans blue that crossed the BBB to stain the cerebrum blue compared to the control group, and that malvidin could mitigate this process in a dose-dependent manner (Figure 1K). Confocal observation of cerebrum tissue cryosections revealed that LPS injection induced a large amount of evans blue across the BBB showing a red fluorescence signal with significantly enhanced fluorescence density compared to the control group, while malvidin could alleviate this process in a dose-dependent manner (Figure 1L). These results suggest that malvidin presents a protective effect in the SAE model induced by LPS injection.

### Malvidin prevents cerebrum mitochondrial dysfunction and ROS accumulation in response to LPS stimulation

**FIGURE 2:**
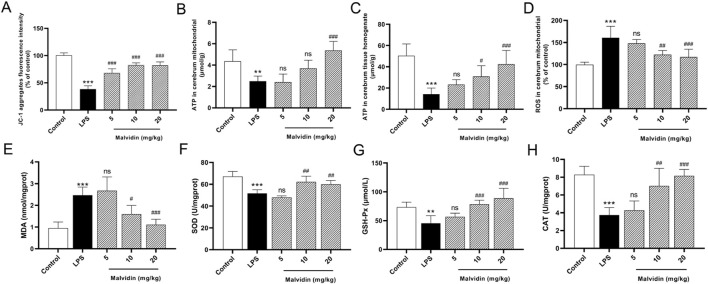
Roles of malvidin on LPS-induced mice cerebrum mitochondrial dysfunction and ROS accumulation. **(A)** JC-1 aggregates fluorescence intensity in the purified cerebrum mitochondrial was detected using mitochondrial membrane potential assay kit (*n* = 6). **(B, C)** ATP contents were measured both in the purified cerebrum mitochondrial and cerebrum tissue homogenates using ATP content assay kit (*n* = 6). **(D)** ROS levels were detected in the purified cerebrum mitochondrial using ROS assay kit with fluorescent probe H_2_DCFDA (*n* = 6). The MDA contents **(E)**, SOD activities **(F)**, GSH-Px activities **(G)**, CAT activities **(H)** in cerebrum tissue homogenates were measured using biochemical kits (*n* = 6). * represents comparison between LPS group and control group, whereas # indicates analysis among LPS group and malvidin pretreatment groups. # is *p* < 0.05, **/## is *p* < 0.01, ***/### is *p* < 0.001, and ns is *p* > 0.05.

Mitochondrial dysfunction is an important causative factor in sepsis-induced multiorgan failure, including SAE (Haileselassie et al., 2020). To explore the protective mechanism of malvidin in SAE mice, we first evaluated its effects on cerebrum mitochondrial function. Mitochondrial membrane potential (MMP) is an important physiological indicator of mitochondria. JC-1, a fluorescent probe, is used to detect mitochondrial membrane potential through distinguishing aggregates in the mitochondrial matrix under high membrane potential or monomers under low membrane potential. In this study, LPS injection significantly decreased the fluorescence density of JC-1 aggregates in purified cerebrum mitochondria, while the fluorescence density of JC-1 aggregates gradually enhanced with increasing doses of malvidin treatment (Figure 2A). These data indicates that the electron transport chain in mitochondria is disturbed, resulting in mitochondria in a stressful state reducing the MMP levels. Mitochondria are important sites for intracellular ATP production. To further assess mitochondrial function, we measured the levels of ATP in purified cerebrum mitochondria and in brain tissue homogenates, respectively. The results showed that ATP contents were significantly reduced both in the purified cerebrum mitochondria and cerebrum tissue homogenates, while malvidin reversed ATP levels, suggesting that malvidin alleviated cerebrum mitochondrial dysfunction (Figures 2B, C). Mitochondria are important sites for cellular ROS production, however, mitochondrial dysfunction may trigger ROS accumulation. In our study, LPS injection led to a large accumulation of ROS in the cerebrum, while malvidin enhanced the ROS scavenged ability with increasing treatment dose (Figure 2D). Consistent with ROS levels, LPS injection caused up-regulation of MDA contents and decline of SOD, GSH-Px, CAT, while malvidin decreased lipid peroxidation MDA and increased antioxidant enzymes of SOD, GSH-Px, CAT (Figures 2E–H), illustrating that malvidin prevents LPS induced redox imbalance and ROS accumulation in the cerebrum. Overall, these results suggest that malvidin protects SAE by preventing from cerebrum mitochondrial dysfunction and ROS accumulation.

### Up-regulation of uncoupling protein 2 (UCP2) in LPS stimulated mice cerebrum and BV-2 cells after malvidin treatment

**FIGURE 3:**
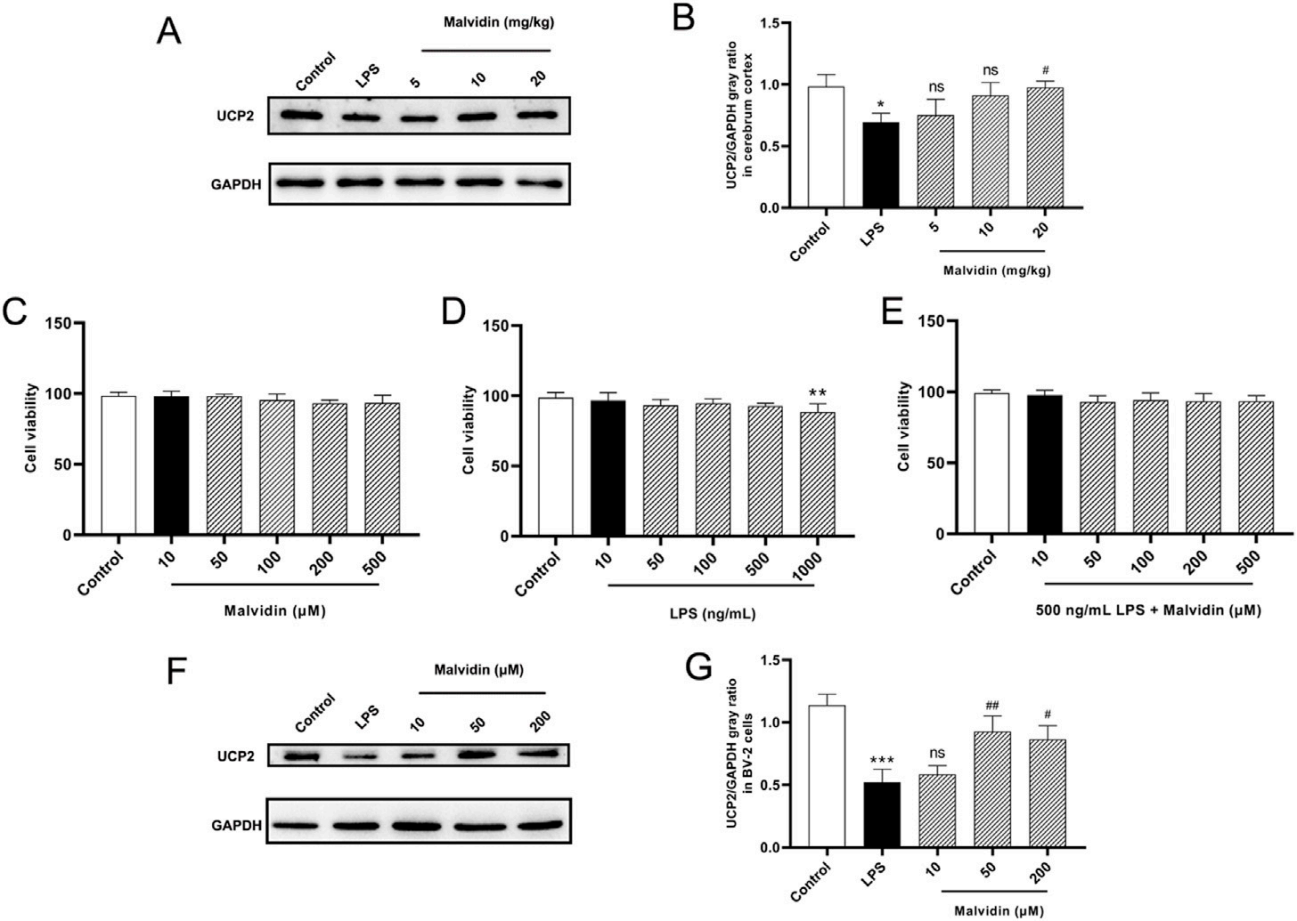
Malvidin enhanced UCP2 protein expression in SAE mice cerebrum and LPS stimulated BV-2 cells. **(A)** UCP2 protein expression levels in the cerebrum tissues were detected using immunoblotting assays. GAPDH was used as an internal reference. **(B)** Gray ratio analysis of UCP2/GAPDH in **(A)** using ImageJ and excel software (*n* = 3). **(C)** Cell viability determination of BV-2 after stimulation with malvidin (10, 50, 100, 200, 500 μM) for 24 h using CCK8 assays. **(D)** Cell viability determination of BV-2 after stimulation with LPS (10, 50, 100, 500, 1000 ng/ml) for 24 h using CCK8 assays. **(E)** Cell viability determination of BV-2 after co-stimulation with 500 ng/ml LPS and malvidin (10, 50, 100, 200, 500 μM) for 24 h using CCK8 assays. **(F)** Immunoblotting assays detection of UCP2 protein expression levels in the BV-2 cells incubated with or without malvidin (50, 100, 200 μM) for 1 h followed by stimulation with 500 ng/ml LPS for 24 h. **(G)** Gray ratio analysis of UCP2/GAPDH in **(F)** using ImageJ and excel software (*n* = 3). * represents comparison between LPS group and control group, whereas # indicates analysis among LPS group and malvidin pretreatment groups. */# is *p* < 0.05, **/## is *p* < 0.01, ***/### is *p* < 0.001, and ns is *p* > 0.05.

UCPs are mainly located in the inner membrane of mitochondria, transporting mitochondrial metabolites and controlling intracellular ROS homeostasis (Brand and Esteves, 2005; Krauss et al., 2005; Zhang et al., 2017). UCP2 is abundantly expressed in neurons and immune cells (Mattiasson and Sullivan, 2006; Degasperi et al., 2008). Based on these background of UCPs and UCP2, we examined the expression of UCP2 in the cerebrum of SAE mice with or without malvidin treatment. As shown in Figures 3A,B, LPS injection caused a significant downregulation of UCP2 protein expression in the cerebrum of SAE mice compared with the cerebrum of control mice. In contrast, malvidin treatment alleviated this process and dose-dependently increased UCP2 protein expression levels. In addition, we established an LPS-stimulated brain immune cell BV-2 infection model and firstly screened the cell viability under different concentrations of malvidin, LPS, LPS and malvidin co-incubation of BV-2 cells, and determined that malvidin in the range of 10–500 μM and LPS in the range of 10–500 ng/ml had no significant effect on BV-2 cell viability, but 1000 ng/ml LPS stimulation lowered the cell viability (Figures 3C,D). Then, BV-2 cells were co-incubated with 500 ng/ml LPS and different concentrations of malvidin, and the cell viability was not influenced within the range of 10–500 μM (Figure 3E). Therefore, we selected 10, 50 and 200 μM malvidin to pretreat BV-2 cells for 1 h and then added 500 ng/ml LPS to detect the cellular UCP2 protein expression. The results showed that LPS stimulation of BV-2 cells decreased UCP2 protein expression and the addition of different concentrations of malvidin treatment could increase UCP2 protein expression in a dose-dependent manner (Figures 3F,G). These results suggest that UCP2 protein expression is damaged by LPS stimulation but elevated both *in vivo* and *in vitro* SAE mice models treated with malvidin.

### Inhibition of UCP2 restricts the protective effects of malvidin on LPS stimulated mice cerebrum and BV-2 cells mitochondrial dysfunction and ROS accumulation

**FIGURE 4:**
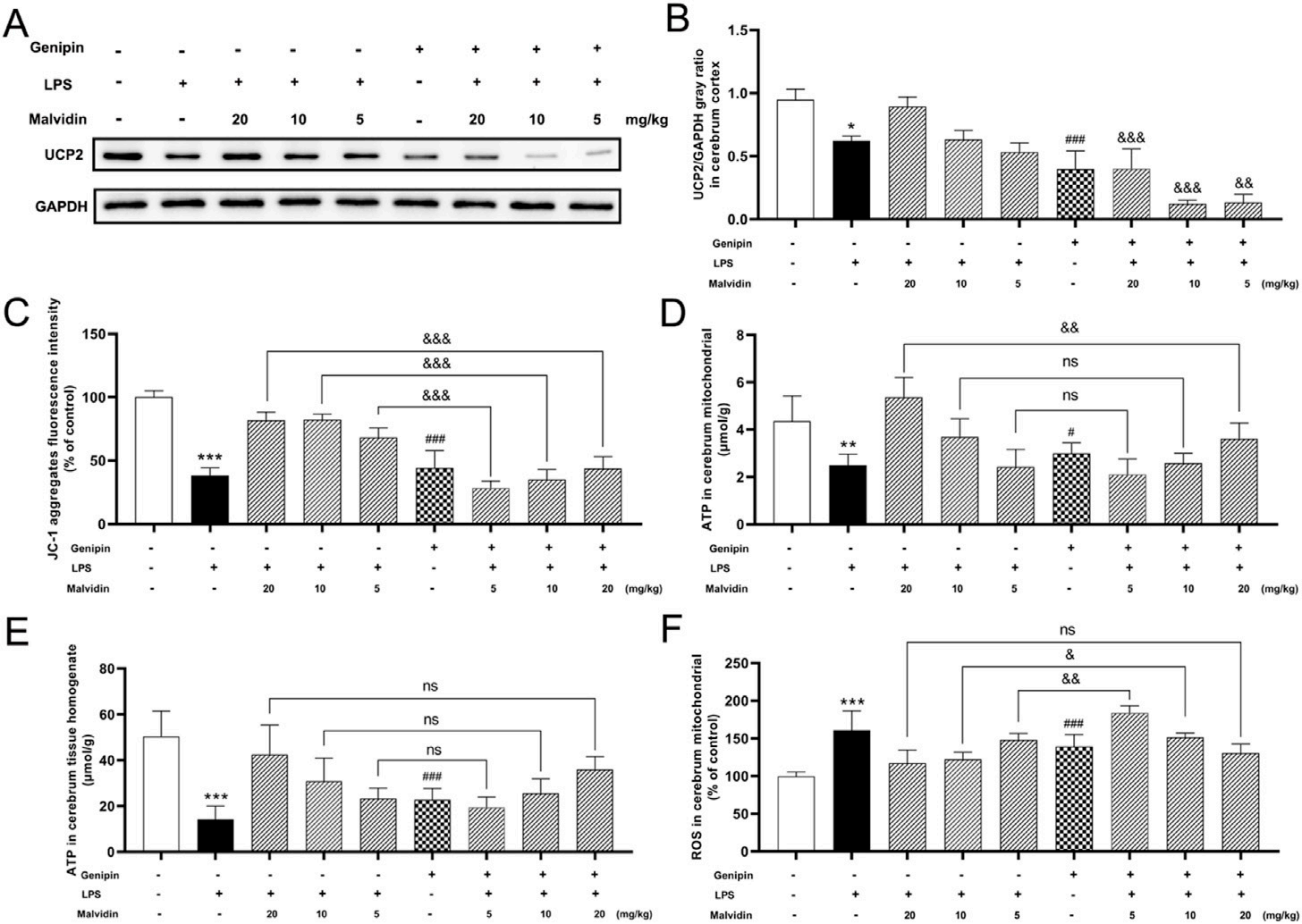
Inhibition of UCP2 on MMP changes, ATP production, and ROS release in malvidin pretreated LPS stimulated mice. Mice were gavaged with genipin prior to LPS injection. **(A)** UCP2 protein expression levels in the cerebrum tissues were detected using immunoblotting assays. GAPDH was used as an internal reference. **(B)** Gray ratio analysis of UCP2/GAPDH in **(A)** using ImageJ and excel software (*n* = 3). **(C)** JC-1 aggregates fluorescence intensity in the purified cerebrum mitochondrial was detected using mitochondrial membrane potential assay kit (*n* = 6). **(D, E)** ATP contents were measured both in the purified cerebrum mitochondrial and cerebrum tissue homogenates using ATP content assay kit (*n* = 6). **(F)** ROS levels were detected in the purified cerebrum mitochondrial using ROS assay kit with fluorescent probe H_2_DCFDA (*n* = 6). * represents comparison between LPS group and control group, # represents comparison between genipin group and control group, whereas & indicates analysis among LPS injection combined with malvidin treatment groups with or without genipin gavage groups. */& is *p* < 0.05, **/&& is *p* < 0.01, ***/###/&&& is *p* < 0.001, and ns is *p* > 0.05.

**FIGURE 5:**
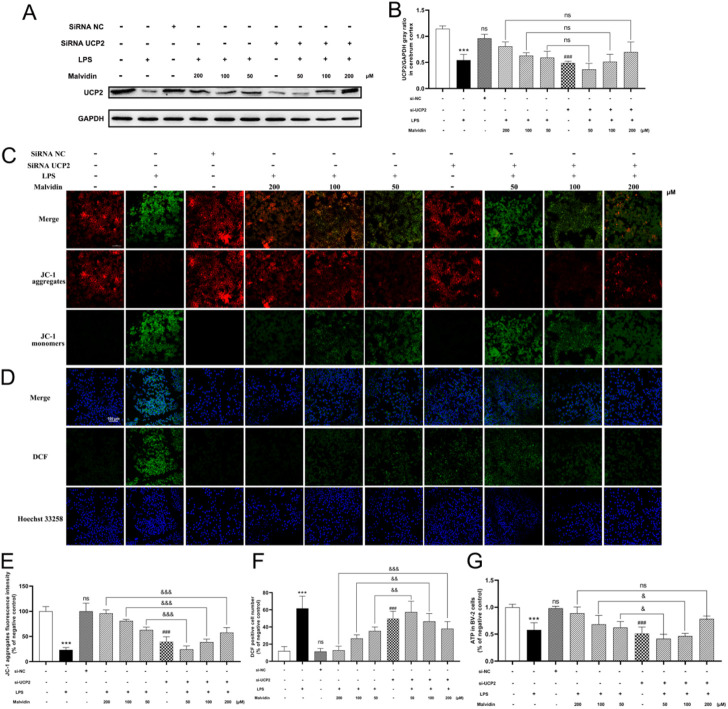
Inhibition of UCP2 on MMP changes, ATP production, and ROS release in malvidin pretreated LPS stimulated BV-2 cells. BV-2 cells were transfected with si-UCP2 to inhibit UCP2 protein expression. **(A)** UCP2 protein expression levels in BV-2 cells were detected using immunoblotting assays. GAPDH was used as an internal reference. **(B)** Gray ratio analysis of UCP2/GAPDH in **(A)** using ImageJ and excel software (*n* = 3). **(C,E)** LSCM observation of JC-1 aggregates fluorescence signals (red) and monomers fluorescence signals (green) in BV-2 cells. The fluorescence density of JC-1 aggregates were analyzed using ImageJ software (*n* = 3). **(D,F)** ROS levels were detected in BV-2 cells using ROS assay kit with fluorescent probe H_2_DCFDA and analyzed using LSCM. The DCF positive signals were made statistical analysis (*n* = 3). **(G)** ATP production was measured in BV-2 cells using ATP content assay kit (*n* =3). Statistical analysis was conducted using one-way ANOVA followed by Tukey’s *post hoc* testing. * represents comparison between LPS group and control group, # represents comparison between si-UCP2 group and control group, whereas & indicates analysis among LPS injection combined with malvidin pretreatment groups with or without si-UCP2 interfering groups. & is *p* < 0.05, && is *p* < 0.01, ***/###/&&& is *p* < 0.001, and ns is *p* > 0.05.

Occurrence of up-regulation of UCP2 in malvidin treated SAE mice models *in vivo* and *in vitro* suggested that UCP2 may be the key treated target of malvidin in SAE. To confirm this suppose, we first inhibited UCP2 *in vivo* through using genipin. The inhibition effects were verified through immunoblotting detection of the UCP2 protein expression in cerebrum. As shown in Figures 4A, B, single genipin gavage treatment led to a significant reduction of UCP2 protein expression compared to the control. Moreover, genipin gavage prior to LPS injection in each malvidin treatment group all triggered decreased UCP2 protein expression levels in cerebrum than that without genipin gavage mice, suggesting genipin successfully inhibited UCP2 protein expression *in vivo*. Next, the effects of UCP2 on malvidin regulated MMP changes and ATP production was determined through measuring the JC-1 aggregates fluorescence intensity and ATP release. Genipin gavage prior to LPS injection in each malvidin treatment group all triggered decreased JC-1 aggregates fluorescence intensity and ATP production (Figures 4C–E), indicating that UCP2 was an important target of malvidin in regulating SAE mice mitochondrial dysfunction. In addition, genipin gavage prior to LPS injection in each malvidin treatment group all exacerbated ROS accumulation (Figure 4F), suggesting that inhibition of UCP2 disrupted malvidin protective roles in alleviating LPS induced ROS accumulation.

si-UCP2 interfere assays were carried out to inhibit UCP2 expression in BV-2 cells. The interfere efficiency of UCP2 was confirmed by immunoblotting assays (Figures 5A, B). LSCM results showed that most JC-1 were existed in aggregates form (red signals) in the control, while LPS stimulation caused the conversion of JC-1 to monomers (green signals). Interfering UCP-2 prior to LPS stimulation in each malvidin treatment group caused the conversion of JC-1 to monomers compared to those without siRNA UCP2 treatment (Figures 5C,E). The influence of UCP2 on ROS release in BV-2 cells were evaluated using H_2_DCFDA probe. LSCM results exhibited that interfering UCP2 expression could enhance the ROS accumulation in malvidin pretreated LPS stimulated BV-2 cells (Figures 5D,F). Also, interfering UCP2 expression reversed the protective roles of malvidin in enhancing the ATP production in LPS stimulated BV-2 cells (Figure 5G). Altogether, these data illustrate that UCP2 is a target of malvidin in alleviating LPS induced cerebrum and BV-2 cells mitochondrial dysfunction and ROS accumulation.

### Malvidin upregulates UCP2 expression in SAE models through phosphorylation of AMPK-α

**FIGURE 6:**
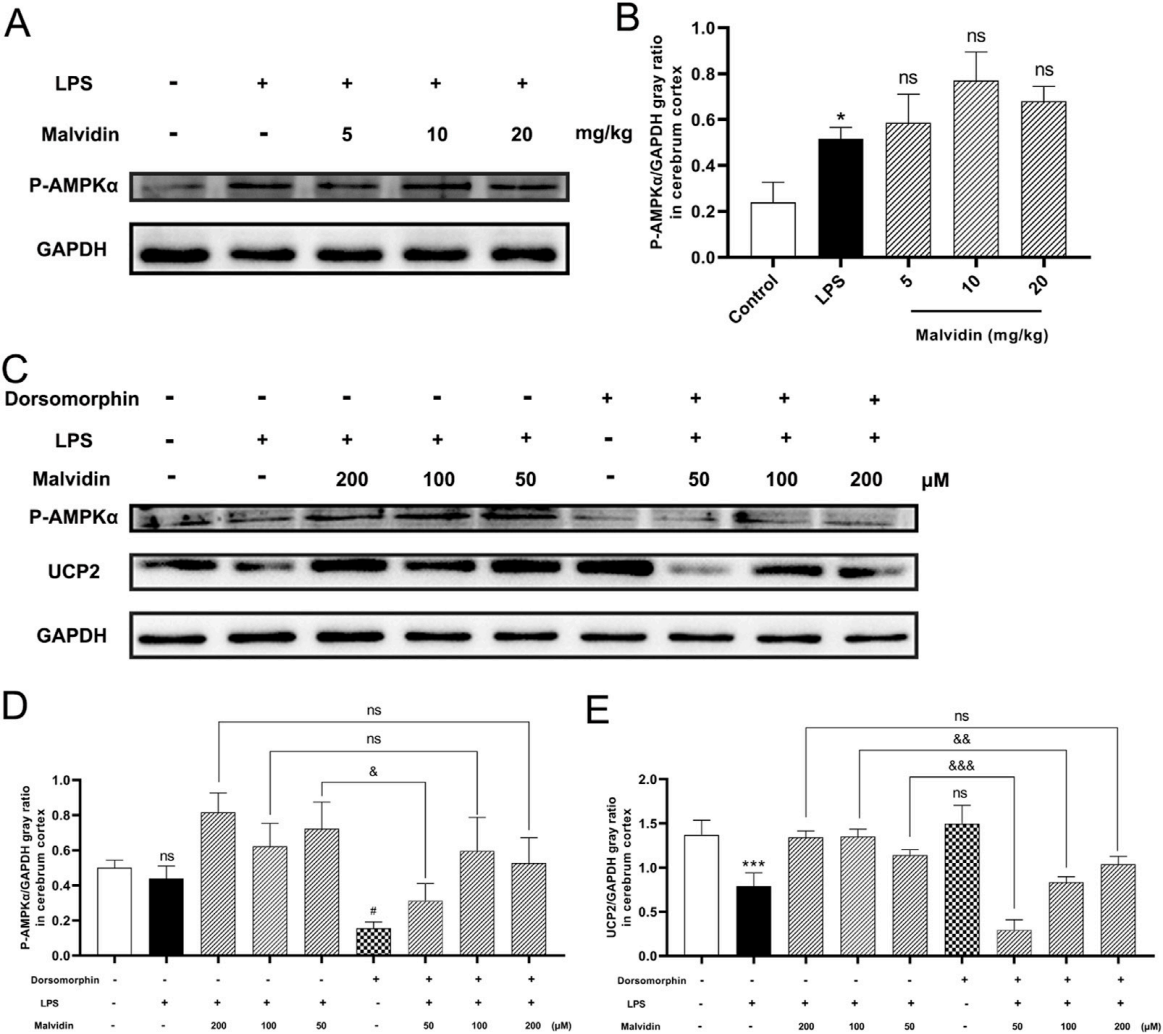
AMPK phosphorylation accelerates UCP2 protein expression in SAE models. **(A)** Phospho-AMPKα protein expression levels in the cerebrum tissues were detected using immunoblotting assays. GAPDH was used as an internal reference. **(B)** Gray ratio analysis of UCP2/GAPDH in **(A)** using ImageJ and excel software (*n* = 3). **(C)** Phospho-AMPKα and UCP2 protein expression levels in BV-2 cells pretreated with or without AMPK inhibitor dorsomorphin. **(D)** Gray ratio analysis of Phospho-AMPKα/GAPDH in **(C)** (*n* = 3). **(E)** Gray ratio analysis of UCP2/GAPDH in **(C)** (*n* = 3). Statistical analysis was conducted using one-way ANOVA followed by Tukey’s *post hoc* testing. * represents comparison between LPS group and control group, # represents comparison among malvidin groups and LPS group in **(B)** or between dorsomorphin group and control group in **(D,E)**, whereas & indicates analysis among LPS injection combined with malvidin pretreatment groups with or without dorsomorphin pretreatment groups in **(D, )**. */#/& is *p* < 0.05, && is *p* < 0.01, ***/&&& is *p* < 0.001, and ns is *p* > 0.05.

AMPK was reported to exerts beneficial effects in sustaining energy homeostasis through suppressing mitochondrial dysfunction triggered ATP excessive consumption (Hardie, 2008) and regulating oxidative stress (Kim et al., 2009). Based on these backgrounds, we hypothesized that malvidin may regulate UCP2 expression through activation of the AMPK signaling pathway, thereby regulating LPS stimulation-induced mitochondrial dysfunction and ROS accumulation. First, we examined AMPK phosphorylation levels in cerebrum tissues and results showed that LPS injection increased AMPK phosphorylation levels in the cerebrum, which were further enhanced by the increased doses of malvidin treatment (Figures 6A,B). To explore the effect of AMPK phosphorylation on UCP2 protein expression, we inhibited AMPK activation *in vitro* and compared the expression of UCP2 in LPS-stimulated BV-2 cells with or without dorsomorphin pretreatment. The results showed that LPS decreased but not significant the AMPK phosphorylation in BV-2 cells, but pretreatment with malvidin enhanced AMPK phosphorylation levels *in vitro*. Pretreatment of BV-2 cells with dorsomorphin successfully down-regulated AMPK phosphorylation levels, but had no effect on UCP2 protein expression levels. Inhibition of AMPK phosphorylation in malvidin pretreated followed by LPS stimulated BV-2 cells down-regulated UCP2 protein expression compared to BV-2 cells without dorsomorphin pretreatment (Figures 6C–E). Overall, these results reveal that malvidin upregulates UCP2 expression through activating AMPK signaling pathway in SAE models.

### Malvidin inhibits pro-inflammatory cytokines release and ROS-mediated NLRP3 inflammasome activation in LPS stimulated mice cerebrum and BV-2 cells

**FIGURE 7:**
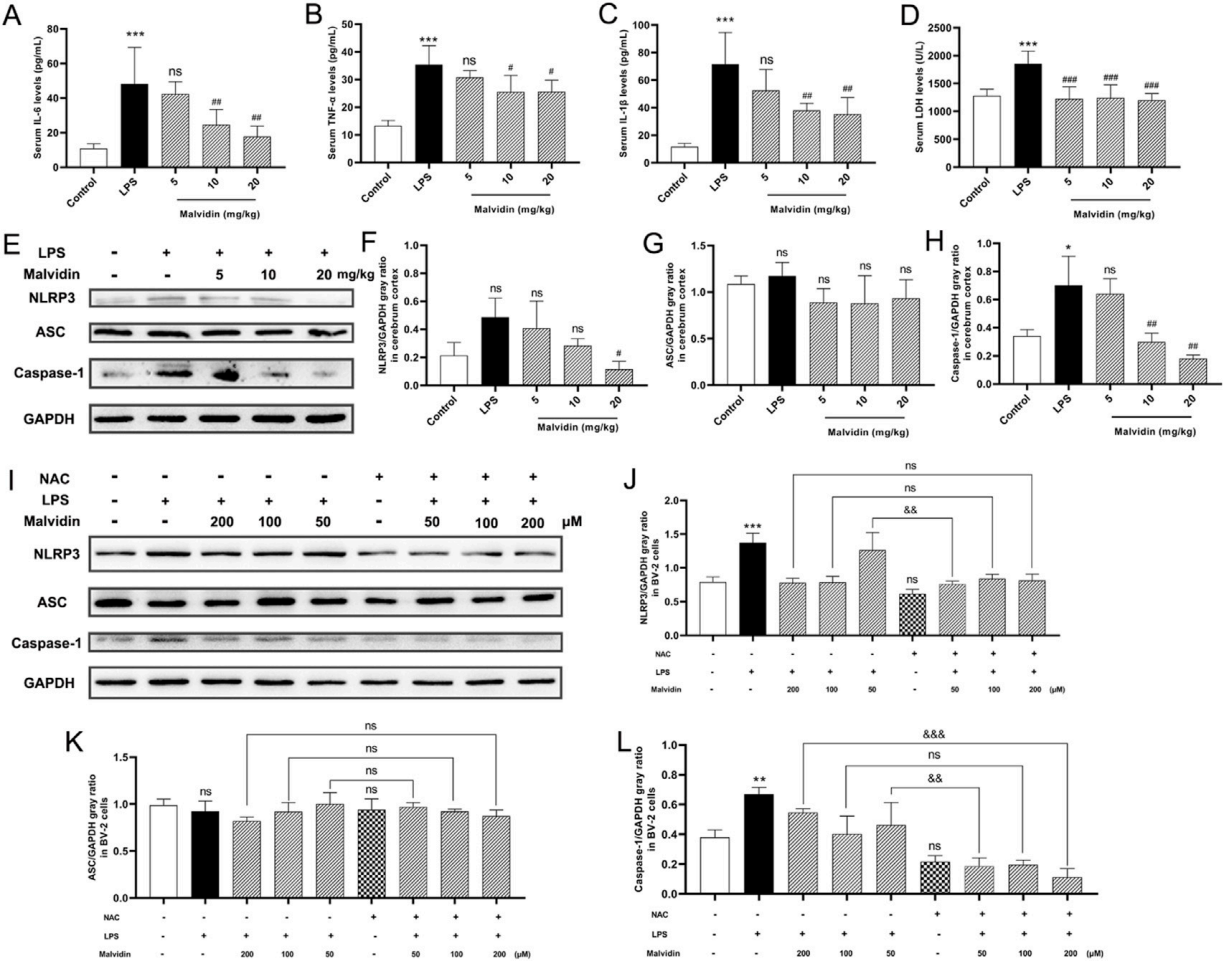
Malvidin protects against LPS induced SAE mice cerebrum inflammation through inhibiting ROS-mediated NLRP3 inflammasome activation. **(A)** Pro-inflammatory cytokines IL-6 **(A)**, TNF-α **(B)**, IL-1β **(C)** secretion and LDH **(D)** release levels in the SAE mice serum. **(E)** The expression levels of NLRP3 inflammasome releated proteins of NLRP3, ASC and caspase-1 in the cerebrum tissues using immunoblotting assays. GAPDH was used as an internal reference. Gray ratio analysis of NLRP3/GAPDH **(F)**, ASC/GAPDH **(G)** and caspase-1/GAPDH **(H)** in **(E)** using ImageJ and excel software (*n* = 3). **(I)** The expression levels of NLRP3 inflammasome releated proteins of NLRP3, ASC and caspase-1 in BV-2 cells lysates using immunoblotting assays. GAPDH was used as an internal reference. Gray ratio analysis of NLRP3/GAPDH **(J)**, ASC/GAPDH **(K)**, and caspase-1/GAPDH **(L)** in **(I)** using ImageJ and excel software (*n* = 3). Statistical analysis was conducted using one-way ANOVA followed by Tukey’s *post hoc* testing. * represents comparison between LPS group and control group, # represents comparison among malvidin groups and LPS group in **(A–D, F–H)** or between NAC group and control group in **(J–L)**, whereas & indicates analysis among LPS injection combined with malvidin pretreatment groups with or without NAC pretreatment groups in **(J–L)**. */# is *p* < 0.05, **/##/&& is *p* < 0.01, ***/###/&&& is *p* < 0.001, and ns is *p* > 0.05.

Sepsis is accompanied with systemic inflammation, which could be transmitted into the central nervous system *via* destroyed blood-brain barrier (Tauber et al., 2021). Thus, we evaluated the protective roles of malvidin in inflammatory cytokines secretion and LDH release. LPS injection caused obviously enhanced pro-inflammatory cytokines IL-6, TNF-α, IL-1β and LDH release in the serum, whereas malvidin alleviated this process (Figures 7A–D). Host NLRP3 inflammasome, a multimeric protein complex, could sense various pathogen associated molecular patterns, endogenous danger-associated molecular patterns as well as ROS, and involves in regulating inflammation (Li et al., 2020). In our study, we found that LPS injection triggered NLRP3 inflammasome key proteins of NLRP3, ASC and caspase-1 upregulation, while malvidin gradually inhibited these three protein expression levels along with the increasing treatment doses in the cerebrum though not significant in the ASC expression (Figures 7E–H).

The above results exhibited that malvidin protected SAE mice through recovering mitochondrial function and inhibited ROS accumulation. Moreover, inflammation-related NLRP3 inflammasome activation could be regulated by ROS (Kelley et al., 2019). Then, whether malvidin protects cerebrum from inflammation in a ROS dependent way. To determine the underlying mechanisms of protective roles of malvidin in SAE models, we pretreated BV-2 cells with or without NAC, a ROS scavenger, prior to malvidin treatment followed by LPS stimulation and intended to explore the roles of ROS in malvidin treated SAE models *in vitro*. As expected, NLRP3 inflammasome key molecules were decreased in the presence of NAC pretreatment compared to the groups pretreatment with malvidin followed by LPS stimulation but without NAC pretreatment especially in the low dose malvidin treatment groups (Figures 7I–L). Overall, these results reveal that malvidin inhibits inflammation SAE models through a ROS dependent manner.

### Malvidin inhibits apoptosis in LPS stimulated mice cerebrum and BV-2 cells in ROS dependent way

**FIGURE 8:**
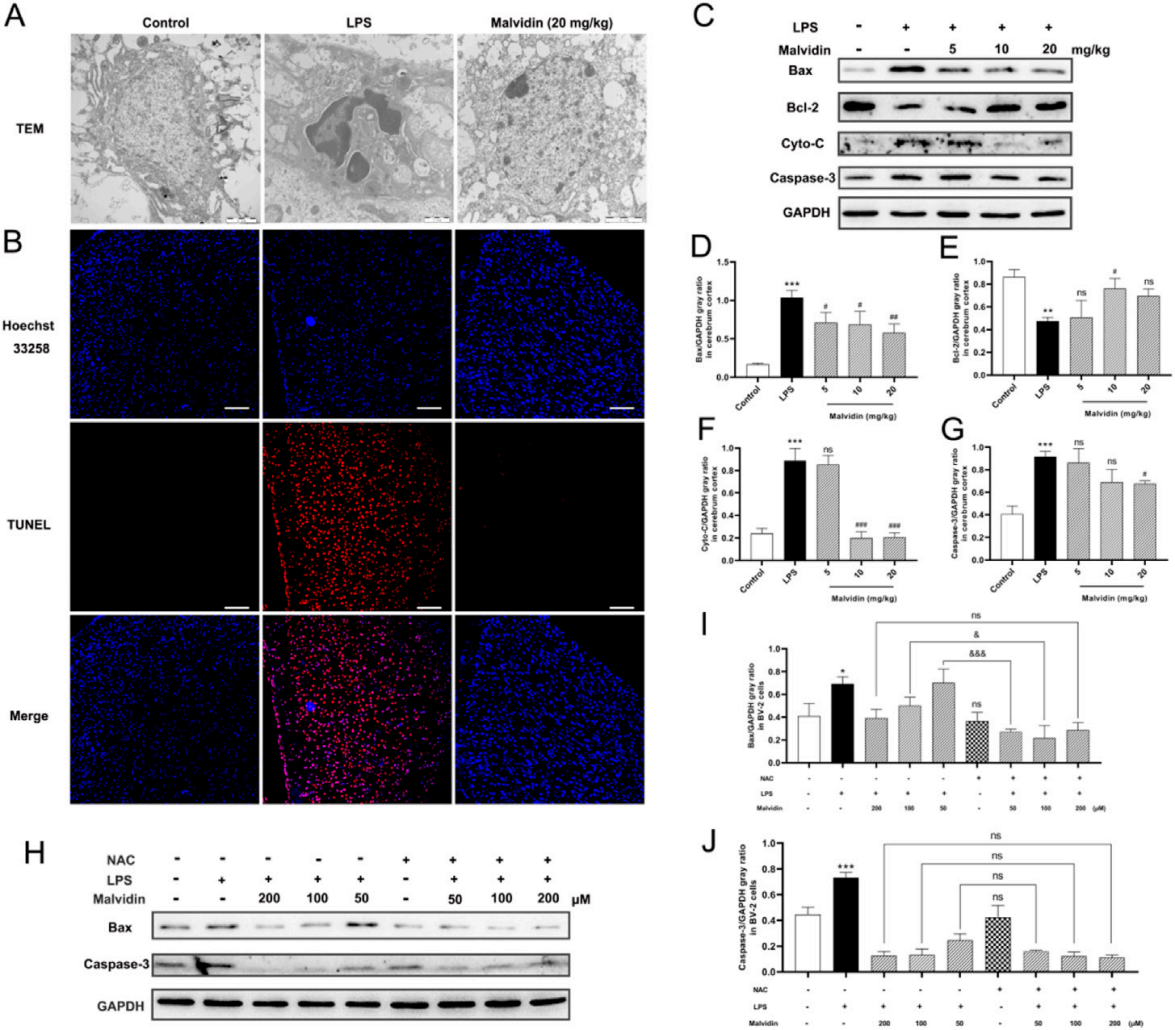
Malvidin protects against LPS induced mice cerebrum injury through inhibiting ROS-mediated apoptosis. **(A)** TEM observation of the microstructures of the nucleus of the cerebral cortex. **(B)** Tunel staining of apoptosis cells in the cerebrum. Scale bar = 100 µm. The tunnel positive cells were stained in red and nuclear is stained with DAPI (in blue). **(C)** The expression levels of apoptosis related proteins of Bax, Bcl-2, Cyto-C and caspase-3 in the cerebrum tissues using immunoblotting assays. GAPDH was used as an internal reference. Gray ratio analysis of Bax/GAPDH **(D)**, Bcl-2/GAPDH **(E)**, Cyto-C/GAPDH **(F)**, caspase-3/GAPDH **(G)** in **(C)** using ImageJ and excel software (*n* = 3). **(H)** The expression levels of apoptosis related proteins of Bax and caspase-3 in the BV-2 cell lysates using immunoblotting assays. GAPDH was used as an internal reference. Gray ratio analysis of Bax/GAPDH **(I)**, and caspase-3/GAPDH **(J)** in **(H)** using ImageJ and excel software (*n* = 3). * represents comparison between LPS group and control group, # represents comparison among LPS injection group and malvidin groups or between NAC group and control group, & indicates comparison among malvidin treatment followed by LPS injection groups with or without NAC pretreatment groups. */#/& is *p* < 0.05, **/## is *p* < 0.01, ***/###/&&& is *p* < 0.001, and ns is *p* > 0.05.

Apoptosis is involved in several organs dysfunction in sepsis-induced injury (Zhang et al., 2019; Ren et al., 2020; Zhou et al., 2020). In this study, TEM results exhibited that, compared with the control group, LPS injection induced nuclear crinkling and chromatin aggregation in the cerebrum. In contrast, apoptosis was significantly improved after high-dose malvidin treatment, and only a small amount of chromatin aggregated at the edge of the nucleus (Figure 8A). TUNEL fluorescence staining results showed that LPS injection caused a large number of cells to undergo apoptosis (red fluorescence signal) and co-localize with the nucleus (blue fluorescence signal), while malvidin treatment significantly inhibited the production of TUNEL-positive apoptotic cells (Figure 8B). We also examined the expression of apoptosis-related proteins, the pro-apoptotic protein Bax, the anti-apoptotic protein Bcl-2, the signature protein cytochrome C (Cyto-C) for altered mitochondrial outer membrane permeability and the apoptosis-activating executive protein caspase-3. The results showed that malvidin elevated LPS-induced Bcl-2 downregulation and decreased LPS-induced upregulation of Bax and Cyto-C expression as well as activation of caspase-3 (Figures 8C–G). These results suggest that malvidin successfully alleviated LPS-induced apoptosis in mice cerebrum and mitigated LPS-induced brain injury by inhibiting apoptosis activated by the mitochondrial pathway.

To further verify the roles of ROS in malvidin treatment model of SAE, we established LPS stimulated BV-2 cells model *in vitro* and analyzed the mitochondrial apoptosis pathway key proteins expression levels in the malvidin treatment followed LPS stimulation infection models with or without NAC pretreatment. NAC did not influence the expression of mitochondrial apoptosis related proteins Bax and caspase-3 but assisted malvidin to decrease their levels especially in the low malvidin dose groups (Figures 8H–J). These results suggest that malvidin inhibits apoptosis in SAE models in ROS dependent way.

## Discussion

Patients with sepsis are often associated with CNS symptoms (Sonneville et al., 2013). Currently, SAE has become a non-negligible factor in increasing mortality in sepsis patients due to its insidious origin and lack of effective diagnostic tools (Pierrakos et al., 2014). The aim of this study is to investigate the pathogenesis of SAE and look for effective prophylactic drugs aiming to provide early clinical intervention for SAE patients to treat brain dysfunction, thereby reducing sepsis mortality and improving the quality of life of SAE patients. Several studies have demonstrated that flavonoids alleviate sepsis brain injury (Zhu et al., 2019), memory impairment (Gong et al., 2019), cognitive dysfunction (Karimian, 2020) by exerting their antioxidant and anti-inflammatory functions. As a member of the flavonoids family, malvidin alleviates inflammation and oxidative stress in several LPS-mediated injury models (Bastin et al., 2020). Similarly, it exerted a protective effect in the SAE model established in this study. Mechanically, malvidin alleviated mitochondrial dysfunction and reduced ROS production by phosphorylating AMPK-α to upregulate UCP2 protein expression, thereby resisting against inflammation and apoptosis. In-depth elucidation of the pathogenic mechanism of SAE and the protective mechanism of malvidin provide a useful reference for clinical development of SAE drugs.

Biomarkers for determining brain injury in SAE include serum NSE and S100β. NSE is a soluble protein mainly found in the CNS, produced specifically by neurons and neuroendocrine cells and increased secretion during brain injury (El Shimy et al., 2018). S100β is a calcium-binding protein produced by glial cells, and its secretion is also increased during brain injury (Yao et al., 2014). The levels of these two proteins in peripheral blood are low, but the levels in serum are significantly increased in the presence of SAE. Therefore, the detection of NSE and S100β levels in serum can assist neurological reflex scoring to reflect the extent of brain tissue damage in SAE. In the present study, we found that LPS stimulation caused a significant increase in serum levels of NSE and S100β while decreasing neurological reflex scoring, indicating that the SAE mice model was successfully established. While malvidin improved neurological reflex scoring and reduced serum levels of NSE and S100β, protecting mice from SAE.

The CNS, as an “immune privilege” organ, is mainly protected by the BBB, which consists of brain endothelial cells and their intercellular tight junctions, intact basement membrane, pericytes, and astrocyte footplate enclosing the glial membrane, and can limit the entry of plasma proteins, inflammatory molecules, and harmful substances in circulating blood into the CNS to maintain the dynamic balance of the internal environment (Hawkins and Davis, 2005). However, the systemic inflammation caused by sepsis leads to activation of brain endothelial cells, which significantly disrupts the integrity of the BBB and causes acute brain dysfunction (Gao and Hernandes, 2021). In the present study, we found that LPS stimulation reduced the expression levels of various TJ proteins and caused brain evans blue leakage, but malvidin upregulated TJ protein levels and alleviated evans blue leakage to protect BBB integrity and permeability in SAE mice.

Several studies have shown that mitochondrial dysfunction is the main cause of neurological dysfunction in patients with sepsis, which eventually progresses to SAE. Physiologically, complexes I and III of the respiratory chain in mitochondria produce small amounts of ROS. However, the inflammatory cytokines that erupt during sepsis can also increase the expression of reduced nicotinamide purine dinucleotide phosphate oxidase and nitric oxide synthase, which in turn initiate oxidative stress and lead to increased ROS production (Quoilin et al., 2014). In the inner mitochondrial membrane, phospholipids are the main components involved in respiratory metabolism and activation of enzymes required in the physiological activities of the membrane ion pump. Increased production of ROS attacks cardiolipin, an important component, and disrupts the activity of related enzymes (Cai et al., 2018). Liaudet et al. (2013) found that increased ROS production in the brain tissue of septic pups led to disruption of mitochondrial membrane lipids and impaired the activity of related enzymes and the structural integrity of the mitochondrial membrane. This resulted in impaired mitochondrial oxidative phosphorylation, which in turn reduced oxygen consumption and decreased the MMP. ATP is an important indicator of mitochondrial function, and mitochondrial dysfunction can lead to a decrease in mitochondrial ATP synthesis, cellular dysfunction, and a decrease in antioxidant synthetic components, leading to a further increase in ROS and ultimately a vicious cycle (Parks et al., 2018). Previous studies indicated that inhibition of oxidative stress and protection of mitochondrial function could improve organ dysfunction caused by sepsis (Yao et al., 2015; Neri et al., 2016). In the present study, we found that LPS stimulation caused decreased MMP and reduced ATP release in purified mitochondria of mice brain, which disrupted mitochondrial function and further promoted the accumulation of ROS in purified mitochondria of brain, accompanied by increased levels of peroxide MDA levels and decreased activities of antioxidant enzymes SOD, GSH-Px, and CAT in brain tissue homogenates, disrupting redox homeostasis and causing oxidative stress. By reversing the alteration of these key indicators, malvidin restored brain mitochondrial function and resisted oxidative stress, and protected mice from SAE.

Determination potential targets that regulate mitochondrial function and ROS production may help to elucidate the pathogenic mechanism of SAE and explore the mechanism by which malvidin exerts its protective effect. As an uncoupling protein closely related to mitochondria, UCP2 has been extensively studied, focusing on its effects on mitochondrial regenerative repair and regulation of membrane potential on the one hand, and on mitochondrial ROS and ATP production on the other. It was demonstrated that UCP2 protected mitochondria and alleviated oxidative stress in LPS-induced renal injury in sepsis (Ding et al., 2019). In our study, we found that LPS stimulation caused a significant decrease in UCP2 expression in the brain tissues of mice, accompanied by a decrease in MMP, a decrease in ATP release and ROS accumulation. In contrast, malvidin treatment caused AMPK-α phosphorylation, upregulated UCP2 expression levels, and alleviated mitochondrial dysfunction and ROS accumulation. *In vivo* blockade of UCP2 using genipin exacerbated mitochondrial dysfunction and ROS accumulation, suggesting that malvidin protects from SAE by targeting UCP2 expression to protect mitochondrial function and ROS homeostasis. Microglia BV-2, an important line of defense for the CNS, are part of the mononuclear phagocyte system and are involved in innate defense mechanisms of the CNS, contributing to tissue repair and recovery (Hanisch, 2002). The integrity of mitochondrial function in BV-2 plays an important role in the function of the CNS. Therefore, we used BV-2 as a model to explore the molecular mechanism of SAE and the protective mechanism of malvidin in SAE. Consistent with the *in vivo* results of SAE, LPS stimulation caused a decrease in UCP2 expression in BV-2 while malvidin reversed this process. To further confirm the function of UCP2, we interfered with UCP2 to inhibit UCP2 expression *in vitro* and transfected an overexpression plasmid to promote UCP2 expression, respectively. In an *in vitro* model of LPS-stimulated BV-2, UCP2 levels positively regulated MMP, ATP release and ROS homeostasis. Dorsomorphin inhibitor blocking phospho-AMPKα assay revealed that malvidin upregulated UCP2 expression by targeting phosphorylation of AMPKα to protect against mitochondrial dysfunction and ROS accumulation in SAE.

In the initial stage of SAE, endotoxin stimulates vascular endothelial cells, disrupts BBB integrity and increases BBB permeability, causing increased synthesis and secretion of various pro-inflammatory cytokines such as TNF-α and IL-6, resulting in an inflammatory storm (Adam et al., 2013). Meanwhile, several studies reported that the overproduction of TNF-α and IL-6 in SAE rats could further lead to other pathological changes such as oxidative stress, neuronal apoptosis, etc, (Calabrese et al., 2007; Azevedo, 2010; Towner et al., 2018). So how does LPS activate the inflammatory storm in SAE model? In our study, we found that LPS stimulation caused elevated levels of NLRP3 inflammasome core proteins expression in brain tissue and BV-2 cells. NLRP3 inflammasome is multi-protein complexes with intracellular pattern-like recognition receptor NLRP3-mediated assembly. Previous studies demonstrated that NLRP3 inflammasome and inflammatory caspase promoted septic heart injury and kidney injury (Deng et al., 2020; Busch et al., 2021). In addition, NLRP3 inflammasome and inflammatory caspase were found to be activated and promote SAE progress (Fu et al., 2019; Xu et al., 2019; Chen et al., 2020). Activation of NLRP3 inflammasome will secrete pro-inflammatory factors IL-1β and IL-18, which regulate the inflammatory response, and ROS accumulation is thought to be an important pathway for NLRP3 inflammasome activation (Jo et al., 2016). In the present study, we found that malvidin alleviated the levels of pro-inflammatory cytokines release from SAE mouse serum, which was accompanied by NLRP3 inflammasome activation. *In vitro* blockade assays using NAC inhibitor further confirmed that malvidin protected against SAE by alleviating NLRP3 inflammasome activation through inhibition of ROS overaccumulation.

Apoptosis is a non-inflammatory cell death involved in sepsis-induced cardiac dysfunction (Zhang et al., 2019) and septic acute kidney injury (Koçkara and Kayataş, 2013). Under physiological conditions, the protein expression of the anti-apoptotic gene Bcl-2 and the pro-apoptotic gene bax are in a relatively stable state, and overexpression of Bax can result in a significant increase in the number of bax/bax homodimers, and an increased cellular responsiveness to death signals, initiating apoptosis (Tsukahara et al., 2006). It was reported that the protein expression of Bcl-2 in peripheral blood was significantly decreased and cells were abnormally apoptotic during sepsis (Chung et al., 2010). Caspase-3 is downstream of the apoptotic cascade and is a common executor of several apoptotic pathways, which can directly cause apoptosis by shearing the structural proteins of the cell (Li et al., 2001). Reduced Bcl-2 can inhibit caspase-3 activation by promoting cytochrome c release and promoting apoptosis (Guo et al., 2008). In the present study, LPS stimulation induced apoptosis in brain tissue of SAE mice, accompanied by activation of endogenous mitochondrial apoptotic pathway, which was alleviated by malvidin. Excess ROS accumulation promotes apoptosis (Wang et al., 2017; Kang et al., 2019). *In vitro* blockade experiments using NAC inhibitor further confirmed that malvidin exerted an antioxidant function to alleviate LPS-mediated SAE apoptosis by inhibiting excessive ROS accumulation, thereby protecting from SAE.

Overall, the present study systematically elucidated the molecular mechanisms of SAE from the perspective of mitochondrial dysfunction and explored the protective role and potential mechanisms of malvidin in SAE. In the LPS-induced SAE model, LPS promoted mitochondrial dysfunction by inhibiting UCP2 protein expression in brain tissue, caused ROS accumulation, mediated oxidative stress, activated NLRP3 inflammasome-mediated inflammatory storm, and also induced apoptosis. By phosphorylating AMPKα, malvidin restored UCP2 protein content in brain, protected mitochondrial function and maintained redox homeostasis, alleviated inflammatory storm by inhibiting ROS-mediated NLRP3 inflammasome activation, and simultaneously inhibited ROS-mediated apoptosis, ultimately protecting the SAE mouse model, restoring neural reflexes, reducing neuronal necrosis, and protecting the BBB integrity and permeability.

## Data Availability

The original contributions presented in the study are included in the article/Supplementary Material, further inquiries can be directed to the corresponding authors.
