# Supplementary material for: Malvidin alleviates mitochondrial dysfunction and ROS accumulation through activating AMPK-α/UCP2 axis, thereby resisting inflammation and apoptosis in SAE mice
Source: Front Pharmacol. 2023 Jan 9;13:1038802. doi: 10.3389/fphar.2022.1038802 (PMC9868257; doi:10.3389/fphar.2022.1038802)
Supplement: Supplementary file 1 [file Table1.DOCX]

**Supplementary TABLE 1 RT-qPCR primer sets information.**

| **Primer name** | **Primer sequences (5’-3’)** |
| --- | --- |
| ZO-1 | Forward: GACTTGTCAGCTCAGCCAGT |
|  | Reverse: GGCTCCTCTCTTGCCAACTT |
| Claudin-5 | Forward: TCTGCTGGTTCGCCAACAT |
|  | Reverse: CGGCACCGTCGGATCA |
| Occludin | Forward: TTTCCTGCGGTGACTTCTCC |
|  | Reverse: GGGGAACGTGGCCGATATAA |
| Cadherin | Forward: GGCTCCGACATCAGGTCTC |
|  | Reverse: CAAGCTGACGCCTACACCTC |
| Actb | Forward: GCCATGTACGTAGCCATCCA |
|  | Reverse: ACGCACGATTTCCCTCTCAG |
